# Coil only middle meningeal artery embolization for the treatment of subdural hematoma: a single center experience

**DOI:** 10.3389/fneur.2026.1902680

**Published:** 2026-07-30

**Authors:** Macy Mitchell, Sarosh Irfan Madhani, Michael Morrison, Mohamed Abouelleil, Matthew Vos, Paul Mazaris

**Affiliations:** 1 Michigan State University College of Human Medicine, Grand Rapids, MI, United States; 2 Department of Neurological Surgery, Corewell Health West, Grand Rapids, MI, United States

**Keywords:** coil embolization, embolization, endovascular coiling, middle meningeal artery, subdural hematoma

## Abstract

**Background and purpose:**

Middle meningeal artery embolization (MMAe) has emerged as an effective adjunct or alternative to surgery for subdural hematoma (SDH). While particles and liquid embolic agents are commonly used, data on standalone coils for embolization remains limited. We aim to evaluate the safety and efficacy of coil only embolization of the middle meningeal artery for the treatment of subdural hematoma in one of the largest single-center series to date.

**Materials and methods:**

We retrospectively reviewed patients who underwent MMA embolization with standalone coils between July 2023 and June 2025. Demographics, procedural characteristics, complications, and radiographic outcomes were collected. Imaging outcomes were assessed at 1 month, 3 months, and 6 months post-procedure. Primary efficacy outcome was unplanned repeat surgical drainage; primary safety outcome was major procedural complications.

**Results:**

Seventy seven patients underwent 101 standalone coil embolizations. Mean age was 71.3 years, and 76.6% were male. Bilateral embolization was performed in 31.2% of cases, and 77.9% of procedures utilized radial access. Technical success was achieved in 100% of cases. Radiographic improvement or resolution was observed in 46.6% at 1 month, 69.3% at 3 months, and 95.1% at 6 months. Four hematomas recurred, with only two requiring repeat surgical drainage. No major procedural complications occurred. Distal coil penetration was associated with higher rates of near-complete or complete resolution, though this was not statistically significant.

**Conclusion:**

Coil only MMAe is a safe and effective treatment for subdural hematoma, demonstrating high technical success, low retreatment rates, and favorable radiographic outcomes. These findings support coils as a viable standalone embolic option.

## Introduction

Subdural hematoma (SDH) has an incidence of 8.2–14.0 per 100,000 individuals, predominantly affecting the elderly (1). The frequency of surgical management of this disease is only expected to increase in the coming years and is projected to be one of the most common neurosurgical procedures by 2030 (2). Traditional treatment involves invasive procedures including surgical burr hole drainage or craniotomy, both of which are effective in the short term but carry notable recurrence rates of 10–30% (3). Meanwhile, middle meningeal artery embolization (MMAe) has garnered attention as an adjunct procedure, aiming to reduce recurrence rates by occluding the MMA and disrupting the vascular supply, targeting neovascularization contributing to the development of SDH, slowing down the accumulation of new blood within the subdural space and ultimately allowing for hematoma resolution (3). Unlike burr-hold drainage, which directly evacuates the hematoma, MMAe targets the underlying vascular supply that contributes to hematoma persistence and recurrence. Several embolization materials have shown promise in treating SDH including polyvinyl alcohol (PVA) particle embolization, liquid embolic agents, (Onyx, NBCA, etc.) and coiling. Coils are detachable platinum-based implants that achieve occlusion via mechanical occlusion of the vessel. Meanwhile, liquid embolic agents transform into a solid agent once injected, providing occlusion. While coil embolization for MMAe has been less extensively studied than its particle and liquid counterparts, it has shown promise in early studies, suggesting both safety and efficacy in standalone and adjunct use. Coiling as an adjunct to other embolization techniques has become standard in some institutions, especially in patients with atypical vascular anatomy deeming distal embolization futile. However, additional data regarding safety and efficacy of treatment with standalone coiling remains valuable (4). Our study adds to the growing body of literature by providing one of the largest series reporting on the outcomes of standalone primary coil embolization for the treatment of subdural hematoma.

## Materials and methods

### Patient selection

We retrospectively reviewed all patients who underwent coil embolization at our institution between July 2023 and June 2025. Only patients who were diagnosed with subdural hematoma and underwent MMAe with standalone coils were included. Patients with acute, subacute, and chronic SDH were eligible for inclusion, although the cohort consisted predominantly of chronic SDH. Patients with bilateral SDH underwent bilateral MMAe and patients with unilateral hematomas had unilateral embolization. Bilateral embolization was performed solely based on hematoma laterality. Of note, most patients had MMAe as an adjunct to surgery. “Standalone coiling” was defined as the use of coils as the sole embolic material during MMAe. Patients who were treated with other embolization techniques or combination materials were excluded from the study. Institutional Review Board approval was obtained prior to beginning the study. The requirement for informed consent for participation in the study was waived secondary to its retrospective design. All procedures were performed as part of routine clinical care and informed consent was obtained prior to operative treatment.

### Data collection

Data was extracted from Electronic Medical Records (EMR) of each patient. Baseline demographic information (age, sex) and information about SDH characteristics was then collected. Procedural information included type of arterial access, fluoroscopy time, and procedural complications. Outcome information was determined using interval head computed tomography (CT) scans in which size of the hematoma and presence of midline shift were reported as continuous variables. Follow up scans were obtained at approximately 1 month, 3 months, and 6 months and outcomes were categorized as resolved, improved, unchanged, or worsened. Radiographic outcomes were categorized based on interval changes in maximal hematoma thickness measured on CT as compared to that of the previous scan. Hematomas were classified as “resolved” when only trace residual hematoma or no visible hematoma remained, “improved” when maximal hematoma thickness had decreased but residual hematoma remained, “unchanged” when there was no meaningful interval change, and “worsened” when the maximal hematoma thickness had increased. While not a validated grading system, this method was applied consistently across all patients at intervals of 1 month, 3 months, and 6 months. Hematoma volume was estimated on CT imaging using the maximum length, width, and height of the hematoma ((X*Y*Z)/2).

In patients with bilateral hematomas, maximal hematoma thickness and hematoma volume were measured independently for each hematoma, with each hematoma treated as a separate lesion for analysis. Midline shift was recorded once per patient as an overall measure of the mass effect and therefore reflected the combined effect of bilateral hematomas when present.

The primary efficacy outcome was the rate of unplanned, repeat surgery secondary to persistent or recurrent hematoma after embolization. The primary safety outcome was the incidence of major complications after embolization.

### Data analysis

Univariate analyses were performed for comparing descriptive characteristics. In normally distributed data, a *t*-test was utilized; in data that is not normally distributed, a Wilcox-sum test was performed. For comparing categorical variables, a Fisher’s exact test was used with small sample size otherwise a chi-square test was performed. Continuous variables were reported as mean with standard deviation and categorical variables reported as frequency. Statistical analysis was performed by statisticians at our institution using R studio (1.1.442). A *p*-value of <0.05 was deemed statistically significant.

### Procedure technique

All procedures were performed by two dual (open and endovascular) trained neurosurgeons. Patients were placed under either moderate or general sedation. Vascular access was obtained via the radial or femoral artery at discretion of the treating neurosurgeon. Under roadmap guidance, the selected catheter was placed over a GLIDEWIRE® (Terumo Interventional, Somerset, NJ, USA) and was ultimately advanced into the external carotid artery. Angiography was performed to visualize the arborization of its branches, including the MMA. The 6Fr RIST™ (Medtronic Neurovascular, Irvine, CA, UA) or Zebra™ (Q’ApelMedical, Inc.) was the most used guide catheter (Envoy, Benchmark, Armadillo were other guide catheters used). A selected microcatheter (Phenom™ 17 or Excelsior XT-17) was then advanced over a microwire, within the guide catheter and advanced into the MMA, beyond the foramen spinosum. Angiography was again performed to visualize the branching pattern of the MMA and identify any dangerous collateral vessels or anastomoses prior to coil placement. The selected number of coils were subsequently placed and detached. All patients had post-coil embolization angiogram to ascertain any anterograde flow as well as characterization of length of embolized artery. Procedures showing no anterograde flow within the MMA were deemed successful.

## Results

A total of 79 patients underwent MMAE for SDH at our institution between July 2023 and June 2025. Two patients received adjunct particle (Onyx) embolization materials and were excluded from analysis. There was a total of 101 embolizations completed within 77 patients. The mean age was 71.3 years and 76.6% of patients were male. 72.3% of cases underwent surgical drainage of SDH in conjunction with MMAe. Patient demographics including age and sex, as well as presenting Glascow Coma Scale (GCS), characteristics of SDH (laterality, maximum thickness, midline shift), prior hematoma evacuation status, and length of admission are detailed in Table 1.

**Table 1:** Patient, hematoma, and treatment characteristics.

Baseline patient characteristics	Mean (SD), Frequency (%), Median [range] Patients, *n* = 77 Embolizations, *n* = 101	*p*-value
Age (years)		
Overall	71.3	
Bilateral	74.7 ± 11.9	0.11
Unilateral	69.7 ± 14	0.11
Gender		
Female	18 (23.4)	
Male	59 (76.6)	
GCS on presentation	15 [10–15]	
Laterality of SDH		
Right	20 (26.0%)	
Left	33 (42.9%)	
Bilateral	24 (31.2%)	
Maximal SDH diameter (mm)	12.8 ± 6.2	
SDH Volume (cm^3^)	52.6 ± 38.1	
Midline Shift (mm)		
Overall	4.09 ± 3.7	
Bilateral	3.13 ± 3.69	
Unilateral	4.9 ± 5.5	
Surgical History		
No history of prior surgical drainage	41 (39.6)	
Prior burr hole drainage	60 (59.4)	
MMAE prior to surgical drainage	13 (12.9)	
Admission status		
Inpatient (n)		
Overall	60 (77.9)	
Bilateral	21 (27.3)	
Length of stay (days)	8.05	
Unilateral	39 (50.6)	
Length of stay (days)	7.67	
Outpatient		
Overall	17 (22.1)	
Bilateral	5 (6.5)	
Unilateral	12 (15.6)	

Bilateral MMAe was performed in 31.2% of cases, corresponding exclusively to patients with bilateral SDH, with a mean fluoroscopy time of 23.8 min for bilateral procedures and 14.4 min for the unilateral procedures. 89.6% of the cases were performed under conscious sedation and 77.9% of procedures were completed with radial access. The median number of coils per embolization was 4. The distance of the distal embolic material within the major branches of the MMA was noted on final angiogram and categorized into proximal, middle, and distal thirds. Further technical specifics are noted in Table 2. One patient developed a femoral pseudoaneurysm treated by vascular surgery; however, no major procedural complications were observed.

**Table 2:** Technical specifications and results.

Procedural specifics	Mean (SD) or *N* (%)	*p*-value
Anesthesia		
Conscious	68 (88.3)	
General	9 (11.7)	
Access		
Radial	60 (77.9)	
Femoral	14 (18.2)	
Both (conversion)	3 (3.9)	
Embolization laterality		
Right	41 (40.6)	
Left	60 (59.4)	
Fluoroscopy time (min)		<0.01
Bilateral	23.8 ± 16.1	
Unilateral	14.4 ± 6.3	
Successful occlusion of target MMA	101 (100)	
Frontal branch embolization		
Proximal	101 (100)	
Middle	41 (40.6)	
Distal		
One branch	41 (40.6)	
Two branches	10 (9.9)	
Parietal branch embolization		
Proximal	100 (100)	
Middle	32 (31.9)	
Distal		
One branch	42 (41.6)	
Two branches	11 (10.9)	
Procedural complications		
Severe	0 (0.0)	
Minor	1 (1.0)	

As detailed in Table 3, the mean length of follow up after embolization was 2.8 months. 46.6% of cases showed resolution or improvement at 1 month, 69.3% of cases showed resolution or improvement at 3 months and 95.1% of cases showed resolution or improvement at 6 months. Four hematomas had recurrence; however, only 2 hematomas required unplanned repeat surgical drainage. Of note, these hematomas were bilateral in a single patient who had unchanged hematoma size with persistent bilateral headaches prompting subsequent burr hole drainage.

**Table 3:** Clinical and imaging outcomes at 1 month, 3 months, and 6 months post MMAE including stratification by extent of most distal embolization.

Outcome	Mean (SD) or *N* (%), *N* = 101^*^
Length of follow up (months)	
Overall	2.80 ± 2.69
Bilateral	3.85 ± 3.70
Unilateral	2.25 ± 1.79
Interval imaging	
1-month imaging (*N* = 82)	
Resolved	4 (4.0)
Improvement	43 (42.6)
Unchanged	26 (26.7)
Worsened	8 (7.9)
3-month imaging (*N* = 48)	
Resolved	6 (5.9)
Improvement	17 (16.8)
Unchanged	21 (20.8)
Worsened	1 (1.0)
6-month imaging (*N* = 33)	
Resolved	3 (3.0)
Improvement	23 (22.8)
Unchanged	6 (5.9)
Worsened	1 (1.0)
12-month imaging (*N* = 13)	
Resolved	1 (1.0)
Improvement	6 (5.9)
Unchanged	6 (5.9)
Worsened	0 (0)
SDH volume at most recent follow up (cm^3^)	13.6 ± 16.7
Stratification by most distal extent	
Complete or near complete resolution	*N* = 26
Limited to middle third	7 (26.9)
Reaches distal third	19 (73.1)
Recurrence	*N* = 4
Limited to middle third	0 (0.0)
Reaches distal third	4 (100.0)
Retreatment	*N* = 2
Limited to middle third	1 (50.0)
Reaches distal third	1 (50.0)
Death or transition to hospice	*N* = 7
Limited to middle third	3 (42.8)
Reaches distal third	4 (57.1)

^*^Percentages for each follow-up interval are based on the number of patients with available imaging at that retrospective time point.

**Figure 1:**
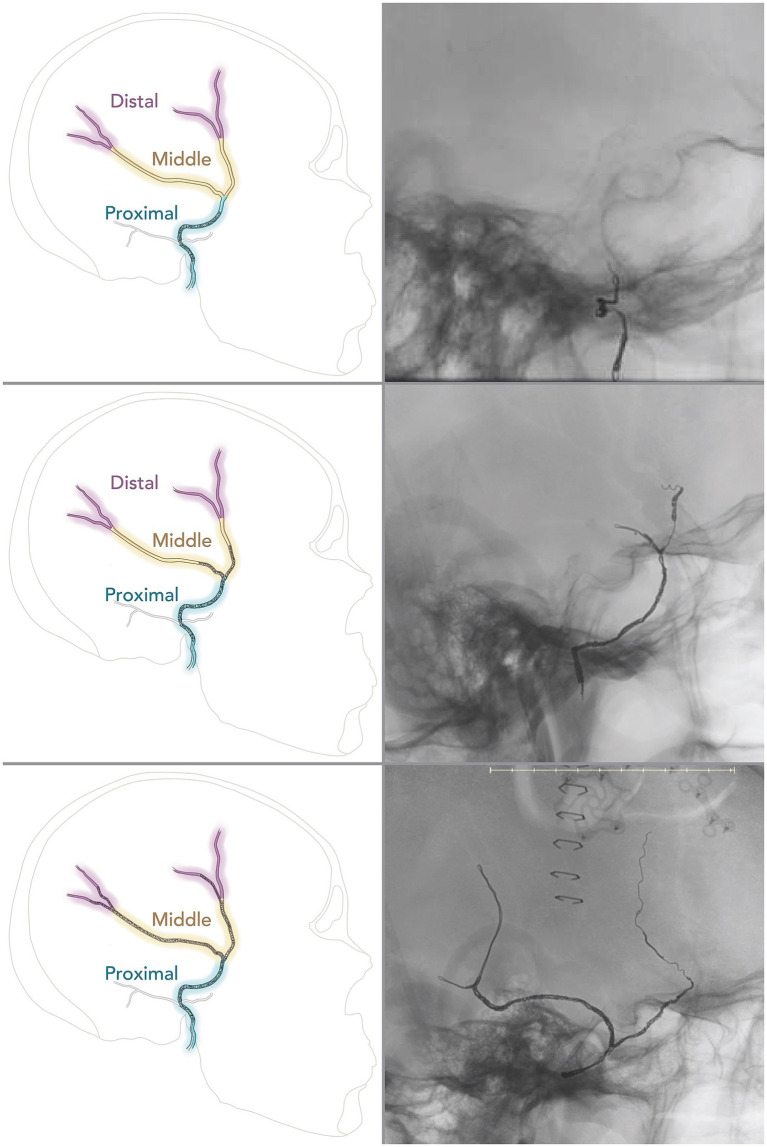
Anatomical classification of the MMA as demonstrated and illustrated in the right lateral projection. The proximal third extends from the MMA origin to its bifurcation into the frontal and parietal branches. The middle third lies between this primary bifurcation and the next bifurcation, while the distal third includes the segments beyond the secondary bifurcation.

A sub analysis was performed comparing imaging outcomes between patients with embolic material limited to the proximal, middle, and distal thirds of the MMA and is illustrated in Figure 1. Classification was based on the angiographic branching anatomy of the MMA. 73.3% of cases had coils embolized into the distal third of at least one branch, while 99.0% were embolized in the middle third of at least one branch. All patients had coils embolized within the proximal third down to the foramen spinosum. There were no delayed procedural complications. Seven patients died following the procedure; 6 of which were unrelated causes, and 1 patient had a repeat fall 3 months after embolization, causing severely worsening hematomas and death. Of note, this patient did have interval changes suggesting re-hemorrhage prior to their fall. Other complications included a seizure in one patient on post-op day 1 and stroke in 1 patient, which was thought to be cardioembolic and unrelated to the patient’s procedure.

Imaging outcomes were stratified by the distal distribution of embolic coils within the MMA (Table 3). Among the 26 patients who achieved complete or near complete radiographic resolution, 73.1% (*n* = 19) had coil deployment extending into the distal third of at least one branch, while 26.9% (*n* = 7) were limited to the middle or proximal third. However, when comparing overall resolution rates across the anatomical tiers, no statistically significant association was found between the depth of the coil penetration and the likelihood of hematoma resolution (*p* = 0.809). Similarly, rates of mortality or transition to hospice did not significantly differ based on the distal extent of embolization.

## Discussion

Seventy seven patients underwent MMA embolization utilizing standalone coils, resulting in 101 embolizations. Complete or near complete resolution was seen in 26 patients on most recent follow up. Of these patients, 12 had complete resolution within 3 months. Complete or near complete resolution was more commonly observed in patients with coil embolization extending into the distal third of at least one MMA branch, and there were no procedural complications. Given that most patients also underwent burr hole drainage, these radiographic outcomes likely reflect the combined treatment approach rather than the isolated effect of coil embolization.

As referenced previously, MMAe is increasingly popular and widely accepted as an effective treatment for SDH. A wide variety of embolic material is currently available for use, however literature on the efficacy of each embolic material, whether used standalone or as combination is scattered. Additionally, current neurovascular literature investigating the use of coiling as an adjunct is limited, with even fewer studies reporting on the use of coiling alone. A study published in 2022 by Khorasanizadeh et al. was the first to report on outcomes in patients treated with coiling as a standalone treatment for SDH. However, out of 94 hematomas, only 12 cases were treated with sole coiling. Of these patients, there were no major treatment related complications, and there was no significant difference in outcomes between standalone coil patients and those treated with adjunct particles (5). While this study promotes the safety and efficacy of standalone coils, it is limited by the small sample size. In 2023, a larger series of 45 patients was published by Iyer et al. on the use of coiling monotherapy with similar results. All patients were successfully treated without any major or minor surgical complications and only 3 patients required retreatment (6). Furthermore, a systematic review published in 2024 composed of 10 studies investigating the use of coils for MMA embolization reported a total of 346 patients, 137 of which were treated with coils alone. They had a cumulative complication rate of 2.3% and rescue treatment was required in less than 10% of patients (3). Most recently, Fecker et al. reported on the use of only coil embolization in 88 patients and found no difference in the technical success rate, need for repeat surgical intervention, or complication rate when compared to other embolic materials (7). Our study adds to the growing body of literature by reporting similar outcomes after 101 embolization in 77 patients, further supporting the safety profile and technical feasibility of coil only embolization; however, as most patients had concomitant surgical evacuation, the efficacy of independent standalone coil embolization cannot be determined from this study.

As this treatment progresses, further research is required to best understand the optimal intricacies of coiling itself as the best location for coils has had conflicting early literature. A large multicenter cohort published in 2023 consisting of 530 patients suggested that regardless of embolic material, targeting the main trunk over the branches promoted better devascularization (3, 8). Meanwhile, a study published in 2024 found that distal penetration of the branches was an independent predictor of rapid hematoma resolution (3, 9). We explored this further in our study by characterizing distal embolization within the MMA using a predefined anatomical categorization (Figure 1). We found that of the patients with near or complete resolution at most recent follow up, they were more likely to have coils present in the distal third of the MMA; however, distal embolization was also present in the small number of recurrent hematomas. Because distal embolization was performed in the majority of cases and there was no statistical significance, these findings remain descriptive rather than indicative of a causal relationship, Larger prospective studies are needed to better define the relationship between embolization extent and clinical outcomes.

The published literature suggests a favorable safety profile of coil only embolization. Interestingly, one of the complications noted for other embolization techniques is distal embolization, whether within the MMA or in non-target arteries. This is particularly dangerous in patients with atypical MMA branching patterns or the presence of petrous or ophthalmic collaterals. While these anomalies are less common, variations in branching patterns, anastomoses, and origination are not inconsequential, as origination of the MMA from the ophthalmic artery has been shown in up to 0.5% of patients (10). Additionally, there is growing evidence supporting a higher prevalence of anastomoses and collaterals than previously considered. In their 2025 study, Fecker et al. noted collaterals or dangerous anastomoses in nearly 40% of their cohort (7). Coiling embolization may be superior for patients with unfavorable anatomy as it theoretically eliminates the risk of embolic materials migrating and causing ischemia in high morbidity regions such as the ophthalmic artery. The reported risk of complications with liquid embolic agents, ranging from 2.2 to 16.7%, with blindness being the most common of severe periprocedural complications (11) whereas blindness following coiling was never observed in our study. Given that primary coiling removes the risk of non-target embolization, it may be the most appropriate method in patients with complex MMA anatomy and a favorable one in the rest, decreasing the risk of distal ischemia and visual complications while maintaining remarkable efficacy.

Additionally, as with liquid and embolic particles, coils may be placed under conscious sedation and remain a minimally invasive treatment for patients who are not amenable to general anesthesia. As most of the patients in our cohort had conscious sedation, the average fluoroscopy time remained under 30 min, even for bilateral procedures. Furthermore, outcomes with standalone coiling as described in our study are in line with previously published literature and may decrease the complications seen with other materials.

Hernandez et al. recently created an angiographic 0–3 scoring system known as the Patency After Coil Embolization (PACE) Score in attempt to better quantify the degree of residual blood flow through the MMA post-coiling and in turn, the likelihood of resolution of SDH. Their single-center retrospective study revealed that patients with a PACE score of 0 were significantly less likely to develop acute blood products following embolization and had greater resolution of chronic SDH on repeat imaging when compared to patients assigned a PACE score of 1 (12). While still novel, this underlines the utility of a quantitative tool and creates a new area of important prospective research. Our findings support the low risk of acute blood products following coil embolization in patients with a low PACE score; however, a comparative analysis could not be performed as all of the patients in our cohort had a PACE score of 0.

Our study is an important contribution to the current literature as it is one of the largest series of standalone coil embolization for SDH. Importantly, approximately two-thirds of the procedures were performed by a single provider, reducing technical variability and allowing for consistency amongst results. As detailed above, the procedure requires minimal equipment and was achieved with radial access in the large majority of cases with a short fluoroscopy time, minimizing radiation exposure to both the patient and the operating room staff.

This study is not without limitations. The retrospective nature limits procedure standardization and presents the potential for confounding. The number of coils deployed varied at discretion of the treating neurosurgeon, introducing variability in technique and cost. Some of the follow up data in this study remains incomplete, with several patients awaiting interval imaging. Notably, 72.3% of our patients underwent concomitant burr-hole drainage, limiting our ability to isolate the independent effect of coil embolization. Therefore, the findings should be interpreted as outcomes of the overall treatment strategy in which coils were the only embolic agent, rather than a standalone treatment. Additionally, this study was not designed to compare treatment timing between surgical evacuation and MMAe. Detailed procedural intervals, baseline comorbidities, and interval clinical assessments were not systematically collected and could not be evaluated but would be valuable in future prospective studies. As this is a single center study, generalizability remains limited. Additionally, baseline antiplatelet and anticoagulant medication use was not systematically collected in this study. As these medications may play a role in hematoma recurrence, we emphasize the value in future prospective studies incorporating baseline antithrombotic therapy into their analysis.

In conclusion, MMAe with coiling for SDH demonstrated high technical success and favorable short-term safety outcomes, with only one patient requiring surgical retreatment. Most patients showed radiographic improvement within the first month. This, in conjunction with the low complication rate, supports the efficacy and safety of coil only MMAe as part of the overall treatment strategy for SDH. Continued follow up as well as standardized, prospective studies are necessary to validate these findings.

## Data Availability

The original contributions presented in the study are included in the article/supplementary material, further inquiries can be directed to the corresponding author/s.
